# Death of a Prominent Physician from the Hypodermic Use of Morphine

**Published:** 1871-12

**Authors:** 


### DEATH OF A PROMINENT PHYSICIAN FROM THE HYPODERMIC USE OF MORPHINE.

We have received the following interesting account of the sad death of the late Dr. Stanton, of Pennsylvania, from Dr. J. R. Speer, an eminent medical gentleman of Pittsburgh, who is spending the winter in Atlanta:

ATLANTA, December, 1871.

*Editors Medical and Surgical Journal:*

Your readers will, no doubt, be interested in the following statement, from two respectable physicians, of the circumstances attending the melancholy death of a most worthy and respectable member of the profession—Dr. D. Stanton, of Pennsylvania.

Dr. Stanton (with whom the writer was personally acquainted) was in the prime of life, of robust and manly frame, and, until a few days before his death, was in the enjoyment of excellent health. The erysipelatous affection which showed itself on his face appears to have been slight, and not accompanied with alarming symptoms, as may be inferred from the fact that there was but little chill or fever. The burning sensation on his face was severe, but not accompanied with swelling and vesication. Up to the time the morphine was administered, and for a short time afterwards, he walked his room and engaged in pleasant conversation with his family. The operation was performed by himself, about half-after seven in the evening; by half-after nine, he was so profoundly asleep and insensible that he could not be wakened; and in four hours afterwards, he expired.

The morphine used in this case seems to be fairly chargeable with the fatal and lamentable result, and adds another to the many instances on record showing the necessity of extreme caution in the administration of anæsthetics generally, but more especially by the hypodermic method. They are remedies of great value in relieving pain in various diseased conditions of the system, but their use is not free from peril.

Very respectfully, etc., J. R. SPEER, M.D.

"THE LATE DR. STANTON—THE TRUE CAUSE OF HIS DEATH.—About six weeks ago Dr. Stanton made two post-mortem examination, in doing which he had to handle, for over an hour, decomposing tissue, and inhale the impure air from the opened bodies. No immediate bad result was noticed; but two weeks ago he spoke to his professional friends about it, and said he believed his system had been slightly poisoned, as he had not felt perfectly well since the examinations. Last Friday a small erysipelatous inflammation appeared on his left cheek, accompanied by a slight chill; on Saturday, at five P.M., the erysipelas involved the whole of the cheek and one-half of the nose. There was also a slight fever. The portion of the face involved in the disease was exceedingly tender, and he said the burning in it gave him no rest. As his physician was leaving him, he remarked that he had lost so much sleep for several nights, that he seemed to have got past his rest, and thought it best to take a small injection of morphia to enable him to pass the night comfortably. His physician offered to give him the injection, but he said he would administer it himself, as he did not wish to take it until near bed-time. At this time Dr. Stanton was cheerful, but manifested no excitement. He was not confined to bed, and he walked about the room with almost his usual vigor.

"Dr. Stanton took the morphia between seven and eight P.M., inserting it deeply into the inner part of the arm, near its junction with the shoulder; the dose was about one grain. For a while afterwards he conversed pleasantly with his family, and then went to sleep. About half-past nine P.M. his wife became alarmed at the character of his breathing, and finding it impossible to awake him, sent quickly for neighboring physicians. Medical attendance reached him by ten P.M. His breathing was then stertorous, his heart's action feeble and irregular, his pupils greatly contracted, his extremities cold, and his stupor profound. Every remedy and appliance known to science were promptly used for his relief, but without avail. He continued to sink until half-past one o'clock on Sabbath morning, when he expired.

"In the opinion of those physicians who witnessed this sad death scene, Dr. Stanton's death was caused by the unusually prompt and complete absorption of the morphia, prostrating a system already struggling with cadaveric poisoning.

"Thus perished one of the brightest ornaments of the medical profession—a victim to his professional devotion, and to the remedy he had so often successfully used to relieve the sufferings of his fellow-men.

"D. McKinney, Jr., M.D.

"J. E. Jackson, M.D."

